# Plasma Donor-Derived Cell-Free DNA Levels Are Associated With the Inflammatory Burden and Macrophage Extracellular Trap Activity in Renal Allografts

**DOI:** 10.3389/fimmu.2022.796326

**Published:** 2022-03-21

**Authors:** Luying Guo, Jia Shen, Wenhua Lei, Pengpeng Yan, Meifang Wang, Qin Zhou, Huiping Wang, Jianyong Wu, Jianghua Chen, Rending Wang

**Affiliations:** ^1^ Kidney Disease Center, the First Affiliated Hospital, School of Medicine, Zhejiang University, Hangzhou, China; ^2^ Key Laboratory of Kidney Disease Prevention and Control Technology, Hangzhou, China; ^3^ National Key Clinical Department of Kidney Diseases, Hangzhou, China; ^4^ Institute of Nephrology, Zhejiang University, Hangzhou, China; ^5^ Zhejiang Clinical Research Center of Kidney and Urinary System Disease, Hangzhou, China

**Keywords:** ddcfDNA, kidney transplantation, Banff lesion score, inflammatory infiltrates, macrophage

## Abstract

Recent studies have confirmed the role of plasma donor-derived cell-free DNA (ddcfDNA) as a reliable non-invasive biomarker for allograft injury after kidney transplantation. Whereas the variability of plasma ddcfDNA levels among recipients has limited their clinical use. This study aimed to explore the intrinsic factors associated with plasma ddcfDNA elevation by investigating the impact of Banff lesions and inflammatory infiltrates on ddcfDNA levels in kidney transplant recipients. From March 2017 to September 2019, a total of 106 kidney transplant recipients with matched allograft biopsies were included, consisting of 13 recipients with normal/nonspecific changes, 13 recipients with borderline changes, 60 with T cell-mediated rejection, and 20 with antibody-mediated rejection. Histologic classification was performed according to the Banff 2017 criteria by two experienced pathologists. Plasma ddcfDNA fractions ranged from 0.12% to 10.22%, with a median level of 0.91%. Banff histology subelements including glomerulitis, intimal arteritis, and severe interstitial inflammation were correlated with increased plasma ddcfDNA levels. The inflammatory cell infiltrate in the allografts was phenotyped by immunochemistry and automatically counted by digital image recognition. Pearson correlation analysis revealed a significant positive correlation between macrophage infiltrations in allografts and plasma ddcfDNA levels. Additionally, macrophage extracellular trap (MET) activity was significantly associated with the rise in plasma ddcfDNA levels. Our findings demonstrated that plasma ddcfDNA could reflect the inflammatory state in renal allografts and suggested the potential role of METs in the pathogenesis of allograft injury.

## 1 Introduction

Donor-derived cell-free DNA (ddcfDNA) generated from donor-cell damage or death is an emerging biomarker for monitoring the health status of allografts after solid organ transplantation (1). Studies have documented elevated plasma ddcfDNA fractions in kidney transplant recipients, especially in antibody-mediated rejection (ABMR) (2, 3), and in other cases such as subclinical rejection, borderline changes, all types of T cell-mediated rejection (TCMR), or even histopathological changes like interstitial fibrosis and tubular atrophy (IFTA), recurrent glomerulonephritis, acute tubular necrosis, and infectious diseases (2, 4–6). Notwithstanding that ddcfDNA has been widely accepted as a marker for predicting the presence of ABMR, the plasma ddcfDNA levels vary in kidney transplant recipients of different histopathologies. Huang et al. (7) reported significant heterogeneity in plasma ddcfDNA fraction in patients with ABMR [interquartile range (IQR): 1.10%–1.90%] and TCMR (IQR: 0.19%–1.30%) and those with no rejection (IQR: 0.26%–1.10%). The mechanisms underlying increased plasma ddcfDNA levels remain uncertain. Although the majority of histopathological manifestations differ, allograft injuries caused by alloimmune or non-alloimmune processes may have overlapping Banff lesion phenotypes. Allograft rejections are intrinsically coupled to a series of Banff histopathology subelements (e.g., microvascular inflammation, tubulitis, and interstitial inflammation). Previous studies have documented the association between plasma ddcfDNA levels and the presence of acute and chronic Banff lesions (2, 8). Due to a limited number of rejection events and attendant histopathology subelements, they could not illustrate any potential subelement that might be the major driving factor of the elevation of ddcfDNA.

“ETosis,” generated by neutrophils (9), macrophages (10), mast cells (11), and eosinophils (12), is a distinct type of cell death (13), where extracellular traps (ETs) actively release DNA–histone complexes (14). Neutrophil extracellular traps (NETs) were the first ETs discovered (15), and NETosis has been identified as one source of cell-free DNA (16, 17). However, the presence of neutrophils in allografts after kidney transplantation is scarce; instead, macrophages were reportedly more commonly involved in the disease pathophysiology. Macrophages are vital for maintaining the internal environment by participating in immune surveillance, host immune defense, and tissue remodeling and repair (18). Most importantly, macrophages are capable of producing ETs. In a study by O’Sullivan et al. (19), myeloperoxidase (MPO)-containing macrophage extracellular traps (METs) were detected in 6/10 of biopsies randomly chosen from patients with glomerulonephritis, suggesting the important role of METs in renal injury. In kidney transplantation, the presence of macrophages was reported to be significantly correlated with later allograft loss (20, 21). At present, there is a paucity of data on the relationship between METs within tissues and plasma ddcfDNA in kidney transplant recipients.

In this study, we sought to explore the intrinsic factors of plasma ddcfDNA elevation by investigating the impact of Banff lesions on ddcfDNA levels in kidney transplant recipients. We observed that severe interstitial inflammation, glomerulitis, and intimal arteritis were accompanied by higher plasma ddcfDNA levels. Furthermore, the inflammatory cell infiltration in allograft was phenotyped by immunochemistry and automatically counted by digital image recognition. Through correlation analysis, we found that macrophage infiltrations in allograft were positively correlated with plasma ddcfDNA. The immunofluorescence assay revealed colocalization of CD68 and MPO in biopsy tissues of allografts. Besides, we also found that the activity of METs was positively correlated with plasma ddcfDNA levels, suggesting the potential involvement of METs in the pathogenesis of allograft injury.

## 2 Materials and Methods

### 2.1 Patients

This study was approved by the Research Ethics Committee of the First Affiliated Hospital of School of Medicine, Zhejiang University (Hangzhou, China; Approval Number 2017-642-1) and was carried out in accordance with the principles of the Helsinki Declaration (2000). We performed a single-center retrospective study that included donation of citizen death or living donor kidney transplant recipients with matched allograft biopsies in our hospital from March 2017 to September 2019. The inclusion criteria consisted of the following: 1) patients aged 18 years or above; 2) patients showing a decline of graft function more than 20% from nadir (22). The exclusion criteria consisted of the following: 1) recurrent/*de novo* glomerulonephritis; 2) BK polyomavirus nephropathy and other infectious diseases; 3) chronic calcineurin inhibitor nephrotoxicity; 4) multiorgan or repeated kidney transplantation. Written informed consent was obtained from all patients before the study commenced. Peripheral blood samples were collected from 171 patients before ultrasound-guided allograft biopsy and treatment. Among those who did not meet the inclusion criteria were 12 cases with recurrent/*de novo* glomerulonephritis, 21 with biopsy-proven BK polyomavirus nephropathy, five with possible polyomavirus nephropathy, and six with TCMR along with urinary BK polyomavirus loads ranging from 3 log10 to 4 log10 copies/ml, two simultaneous pancreas and kidney transplant recipients, three repeated kidney transplant recipients, and 16 cases with chronic calcineurin inhibitor nephrotoxicity. A total of 106 kidney transplant recipients were enrolled, consisting of 13 recipients with normal biopsy/nonspecific changes [no rejection (NR)], 13 recipients with borderline changes, 60 TCMRs, and 20 ABMRs.

### 2.2 Blood Collection and ddcfDNA Quantification

The peripheral blood samples (8 ml) were collected simultaneously with the percutaneous allograft biopsy in cfDNA blood collection tubes (Streck, La Vista, NE, USA). Plasma was separated by centrifugation at 1,600 rpm for 10 min. For pathogen detection, 600 μl of supernatant was removed to extract cfDNA using the Circulating Nucleic Acid kit (Cat. No. 55114; Qiagen, Hilden, Germany). The remaining supernatant underwent a second centrifugation for another 10 min at 16,000 rpm, and 1.8 ml of the supernatant was used for cfDNA extraction and ddcfDNA analysis. Library construction, target region capture sequencing, bioinformatics analysis, ddcfDNA quantification, and pathogen detection were carried out as described in our previous study (23).

### 2.3 Histology, Immunochemistry, and Immunofluorescence

Biopsy tissues underwent a routine process that included formalin fixation and paraffin embedment, followed by hematoxylin and eosin, Periodic Acid-Schiff, and Masson’s trichrome staining. Immunochemistry was performed in all biopsies to visualize T lymphocytes (CD3, CD8; ZSGB-BIO, China), B lymphocytes (CD20; ZSGB-BIO, China), and macrophages (CD68; ZSGB-BIO, China). Whole slide images were scanned by a digital microscopic scanner (NanoZoomer 2.0-HT, Japan). Histologic classification was performed according to the Banff 2017 criteria (24). Banff lesions were scored by two experienced pathologists, including interstitial inflammation (i), tubulitis (t), intimal arteritis (v), glomerulitis (g), peritubular capillaritis (ptc), C4d, interstitial fibrosis (ci), tubular atrophy (ct), vascular fibrous intimal thickening (cv), glomerular basement membrane (GBM) double contours (cg), mesangial matrix expansion (mm), arteriolar hyalinosis (ah), hyaline arteriolar thickening (aah), and microvascular inflammation (mvi). Immunofluorescence assay was performed using the following antibodies: CD68 (Abcam), MPO (Proteintech), and Histone H3 (Cell Signaling Technology).

### 2.4 Automated Cell Counting of Immunochemistry Images

#### 2.4.1 Allograft Biopsy Area Measurement

The slide images were first converted into grayscale and binarized with a threshold of 200 for optical character recognition. Connected component analysis was performed to calculate pixel areas of slide images by OpenCV software (version 4.2.0). The connected components less than 10% of the maximum area were filtered, and the remaining components were utilized for pixel area calculation. Then, the pixel area was converted into the actual physical area by 2.3 pixels per micron.

#### 2.4.2 Allograft Biopsy Area Measurement

Training data preparation: Thirty images that contained complete sections were randomly selected, and the glomerulus was contoured by experts using software Label me (version 4.2.9). A deep convolutional neural network (CNN) was adopted to identify glomerulus in immunochemistry images using PyTorch (https://arxiv.org/abs/1912.01703) framework. In this respect, network architecture U2NET (https://arxiv.org/abs/2005.09007) was applied to perform glomerulus segmentation. The whole slide image was segmented to fit the input images (768 × 768). Characteristic diagrams of three channels were defined as 1) pixels in the glomerulus, 2) pixels on the glomerulus contour, and 3) pixels outside the glomerulus. These diagrams were analyzed to compute the cross-entropy loss. The Adam optimizer was employed for model training with initial learning rate of 0.001 for the first 100 epochs and restricted to 0.0001 until 150 epochs finished. The images were converted into smaller sizes (1,024 × 1,024 with step length of 768 × 768) to fit the size of input images. The first characteristic diagram of the model was applied to restore the original image, and the maximum value was characterized as the overlapping area. The characteristic diagram was converted into binary images with a threshold of 0.5, and then connected component analysis was used to identify the contour of the glomerulus. The output of the model marking results into json files. Then, Label me was used for verification and correction.

#### 2.4.3 Cell Segmentation

Difference of Gaussian (DOG) was performed to identify every individual positive staining cell in immunochemistry images. The Gaussian kernel settings were *σ*_1_ = 0.5 and *σ*_2_ = 3. The results of the DOG were converted into binary images with a threshold of 10. Connected components were found, and areas less than 10 were removed. The remaining components were recognized as positive staining cells. One further step was performed to remove false-positive cells. For each pixel in a component, if the maximum RGB value was less than 100, this component was kept or otherwise removed as a false-positive staining cell.

### 2.5 Statistical Analysis

R software (version 3.6.2, 64-bit) was used for statistical analysis in this study. Patient demographics and plasma ddcfDNA levels among subgroups were compared using the chi-square test (binary and ordinal variables) and Kruskal–Wallis rank-sum test (continuous variables) with the Wilcoxon rank sum test (source; “http://www.statmethods.net/RiA/wmc.txt”) as posttest. The ggplot2 package was used for data visualization. The ComplexHeatmap package was applied for hierarchical clustering. Pearson correlation test was applied for correlation analysis. *P* values <0.05 were considered statistically significant.

## 3 Results

### 3.1 Patients and Plasma ddcfDNA Levels

**Figure 1:**
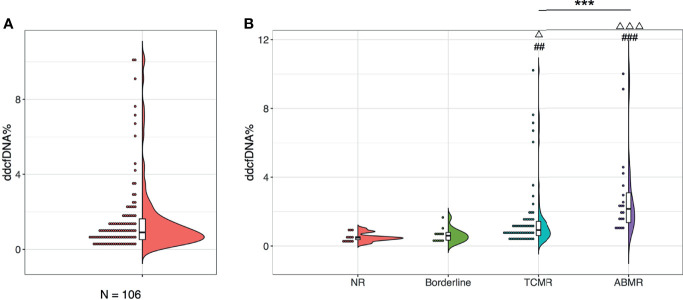
Plasma ddcfDNA levels in kidney transplant recipients of different histopathologies. Distribution of plasma ddcfDNA fractions in total 106 patients enrolled **(A)** and in NR (n = 13), Borderline (n = 13), TCMR (n = 60), as well as ABMR (n = 20) subgroups with different histopathologies **(B)**. Raincloud plot with bold line represents median levels and box indicating the interquartile range. NR, no rejection; Borderline, borderline changes; TCMR, T cell-mediated rejection; ABMR, antibody-mediated rejection; ^##^ *P* < 0.01 and ^###^ *P* < 0.001 compared with the NR cohort; ^△^ *P* < 0.05 and ^△△△^ *P* < 0.001 compared with the borderline cohort. ***P < 0.001.

A total of 106 plasma samples with matched biopsies from 106 kidney transplant patients that underwent ddcfDNA measurement was included in our study. According to the Banff 2017 criteria, 13 recipients were diagnosed with normal biopsy/nonspecific changes (NR) and 13 recipients were with borderline changes, while the remaining 80 recipients suffered from acute rejection, of whom 60 patients were diagnosed as TCMR and 20 patients as ABMR (**Table 1**). Histopathological staining in each allograft biopsy was evaluated by two experienced histologists and classified into Banff lesion scores, including glomerulitis (g), tubular atrophy (ct), tubulitis (t), intimal arteritis (v), interstitial inflammation (i), interstitial fibrosis (ci), and peritubular capillaritis (ptc), as displayed in **Table 2**. Consistent with the histopathological diagnosis, we found a greater proportion of severe Banff lesion scores in TCMR and ABMR subgroups (**Table 2**). The distribution of plasma ddcfDNA fractions in these patients was presented in **Figure 1A**. In all patients enrolled, the plasma ddcfDNA fractions ranged from 0.12% to 10.22% with a median level of 0.91% (0.51%–1.55%, 25% quantile). The ddcfDNA fractions for different histopathological diagnoses, including NR, borderline change, TCMR, and ABMR, are shown in **Figure 1B**. No significant difference in plasma ddcfDNA fraction levels was found between NR (0.48%, 0.35%–0.69%) and borderline (0.60%, 0.34%–0.80%) groups (*P* = 0.608). In the TCMR group, plasma ddcfDNA fractions were more heterogeneous, ranging from 0.26% to 10.22%, and were significantly higher than both NR and borderline groups (*P* < 0.05). The highest plasma ddcfDNA fractions were observed in the ABMR group, with a median of 2.145% (range 0.89%–9.99%). Moreover, the ddcfDNA levels in the ABMR group were significantly higher than those in the NR, borderline, and TCMR groups (*P* < 0.001).

**Table 1:** Patient demographics.

	NR	Borderline	TCMR	ABMR	*P* value
**Donor-associated parameters**					
Donor age	45.42 ± 4.05	41.15 ± 4.26	45.96 ± 1.50	41.58 ± 3.19	0.434
Donor gender	8/5	7/6	44/16	11/9	0.322
Donor Cr	131.00 ± 27.19	73.08 ± 5.15	94.20 ± 7.97	77.20 ± 13.68	0.083
Donor (DCD/LD)	8/5	6/7	41/19	8/12	0.108
**Recipient-associated parameters**					
Age	41.42 ± 3.71	35.00 ± 2.34	39.49 ± 1.52	31.58 ± 2.50	0.038
Gender	11/2	8/5	41/19	16/4	0.433
Height (cm)	167.33 ± 1.69	167.15 ± 2.69	166.33 ± 1.14	168.63 ± 1.56	0.699
Weight (kg)	57.35 ± 2.41	57.92 ± 2.98	61.77 ± 1.76	66.44 ± 3.83	0.189
BMI (kg/m^2^)	20.52 ± 0.93	20.57 ± 0.64	22.16 ± 0.47	23.23 ± 1.19	0.13
HLA-MM	3.50 ± 0.45	2.77 ± 0.38	3.13 ± 0.17	2.84 ± 0.26	0.338
Dialysis					0.379
None	2	0	5	0	
HD	7	8	43	15	
PD	4	5	12	5	
Induction					0.119
None	0	1	10	4	
Simulet	4	6	30	12	
ATG	9	6	20	4	
WBC	8.77 ± 1.83	8.50 ± 1.37	9.41 ± 0.61	8.96 ± 0.85	0.945
Hb	10.78 ± 0.44	11.10 ± 0.92	15.86 ± 2.44	10.63 ± 0.51	0.388
Plt	197.67 ± 13.14	201.08 ± 15.41	208.67 ± 8.52	204.89 ± 17.11	0.986

NR, no rejection; Borderline, borderline changes; TCMR, T cell-mediated rejection; ABMR, antibody-mediated rejection; DCD, donation of citizen death; LD, living donor; HLA-MM, HLA-mismatch; HD, hemodialysis; PD, peritoneal dialysis; ATG, anti-thymocyte globulin; WBC, white blood cell; Hb, hemoglobin; Plt, platelet.

P value represents the difference between NR, borderline, TCMR, and ABMR using one-way ANOVA and chi-square test.

**Table 2:** Banff lesion subelements in each group.

	NR	Borderline	TCMR	ABMR	*P* value
**N**	**13**	**13**	**60**	**20**	
**g**					**<0.001**
g0	7	8	40	5	
g1	4	3	8	1	
g2	2	1	6	4	
g3	0	1	6	10	
**ct**					**0.050**
ct0	6	1	7	1	
ct1	7	10	37	13	
ct2	0	1	11	5	
ct3	0	1	5	1	
**t**					**<0.001**
t0	8	0	4	5	
t1	5	11	5	7	
t2	0	2	29	6	
t3	0	0	22	2	
**v**					**<0.001**
v0	13	13	37	10	
v1	0	0	22	4	
v2	0	0	1	6	
**i**					**<0.001**
i0	8	0	3	3	
i1	5	11	8	4	
i2	0	1	25	6	
i3	0	1	24	7	
**ci**					**0.003**
ci0	11	6	13	4	
ci1	2	5	19	8	
ci2	0	1	14	5	
ci3	0	1	14	3	
**ptc**					**0.002**
ptc0	10	12	39	7	
ptc1	1	0	7	1	
ptc2	2	1	3	8	
ptc3	0	0	11	4	

NR, no rejection; Borderline, borderline changes; TCMR, T cell-mediated rejection; ABMR, antibody-mediated rejection; g, glomerulitis; ct, tubular atrophy; t, tubulitis; v, intimal arteritis; i, interstitial inflammation; ci, interstitial fibrosis; ptc, peritubular capillaritis.

P value represents the difference between NR, borderline, TCMR, and ABMR using chi-square test.

### 3.2 Banff Lesions and Plasma ddcfDNA Levels

**Figure 2:**
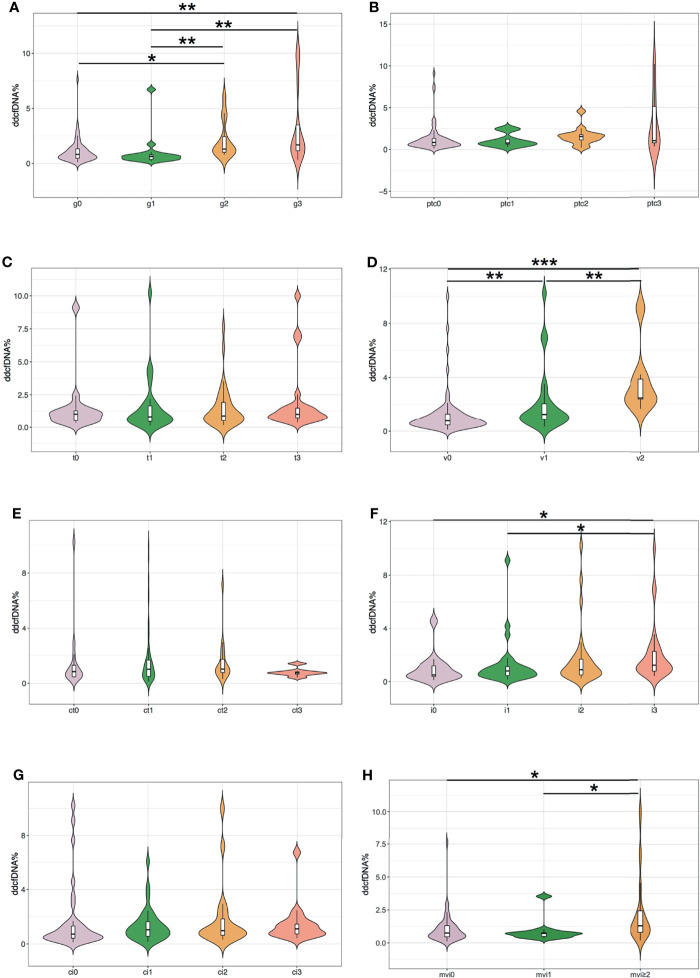
Plasma ddcfDNA fractions in kidney transplant recipients with different Banff lesion scores. **(A)** Glomerulitis (g); **(B)** peritubular capillaritis (ptc); **(C)** tubulitis (t); **(D)** intimal arteritis (v); **(E)** tubular atrophy (ct); **(F)** interstitial inflammation (i); **(G)** interstitial fibrosis (ci); and **(H)** microvascular inflammation (mvi, g+ptc). Violin plot with bold line represents median levels and box indicating the interquartile range. *P < 0.05, **P < 0.01, and ***P < 0.001.

To investigate whether the rise in plasma ddcfDNA fractions were caused by Banff lesions in the allograft, the ddcfDNA levels in matched biopsies with different Banff lesions were further compared. Paired allograft biopsies and plasma ddcfDNA levels were further analyzed to investigate the relationship between Banff lesion subelements and ddcfDNA levels. Banff lesion scores, including glomerulitis (g) and intimal arteritis (v), were associated with elevated plasma ddcfDNA fractions/concentrations, while no significant correlation was found for peritubular capillaritis (ptc) and tubulitis (t) (**Figure 2** and **Supplementary Figure S1**). Increased plasma ddcfDNA levels were accompanied by higher g (*P* < 0.05) and v (*P* < 0.01) scores. The presence of severe interstitial inflammation (i3) was significantly associated with higher plasma ddcfDNA fractions compared with i score ≤1 (*P* < 0.05; **Figure 2F**). Furthermore, moderate to severe microvascular inflammation (mvi score ≥2) and C4d staining, features of ABMR, were related to higher plasma ddcfDNA fractions in recipients (*P* < 0.05; **Figure 2H** and **Supplementary Figure S2F**). Whereas no significant correlation was found between plasma ddcfDNA levels and features of chronic ABMR (cg) or cellular allograft injury (cv, ci, or ct) (**Figure 2G** and **Supplementary Figures S1–S3**). The presence of arteriolar hyalinosis (ah) was associated with decreased plasma ddcfDNA levels (both fractions and concentrations), while no correlation was found for mesangial matrix (mm) and hyaline arteriolar thickening (aah) (**Supplementary Figures S2, S3**).

### 3.3 Inflammatory Infiltrates and Plasma ddcfDNA Fractions

**Figure 3:**
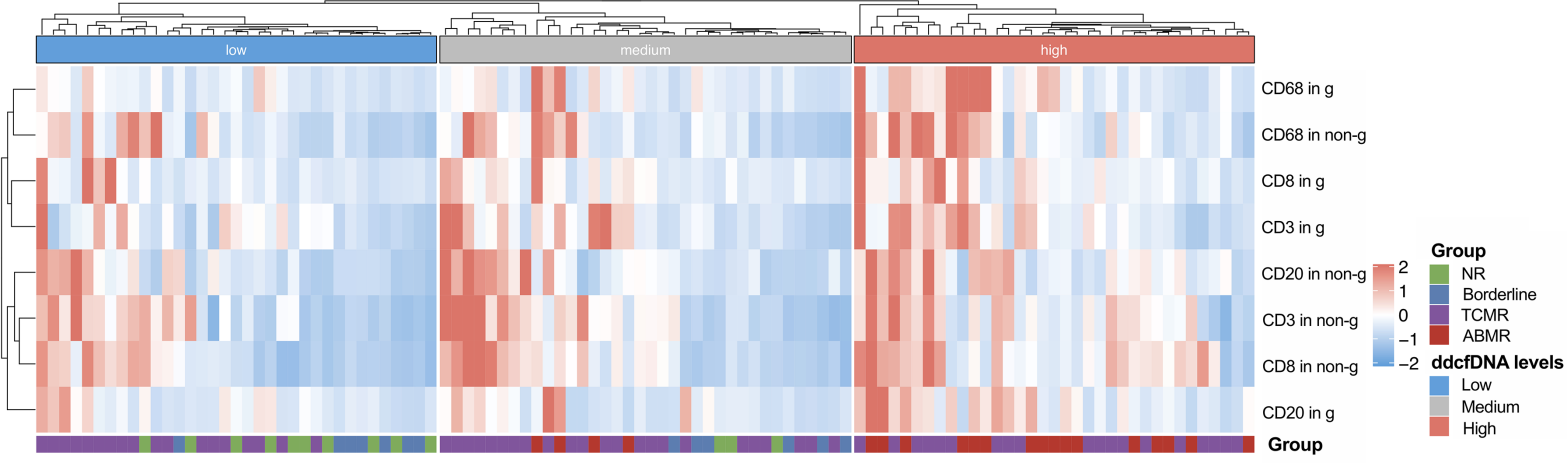
Hierarchical clustering of infiltrated inflammation in each biopsy with different plasma ddcfDNA levels. Heat map showing T cell (CD3+), cytotoxic T cell (CD8+), B cell (CD20+), and macrophage (CD68+) infiltration in glomerular as well as non-glomerular area of low, medium, high levels of plasma ddcfDNA subgroups. Average expression scale is shown on the right.

**Figure 4:**
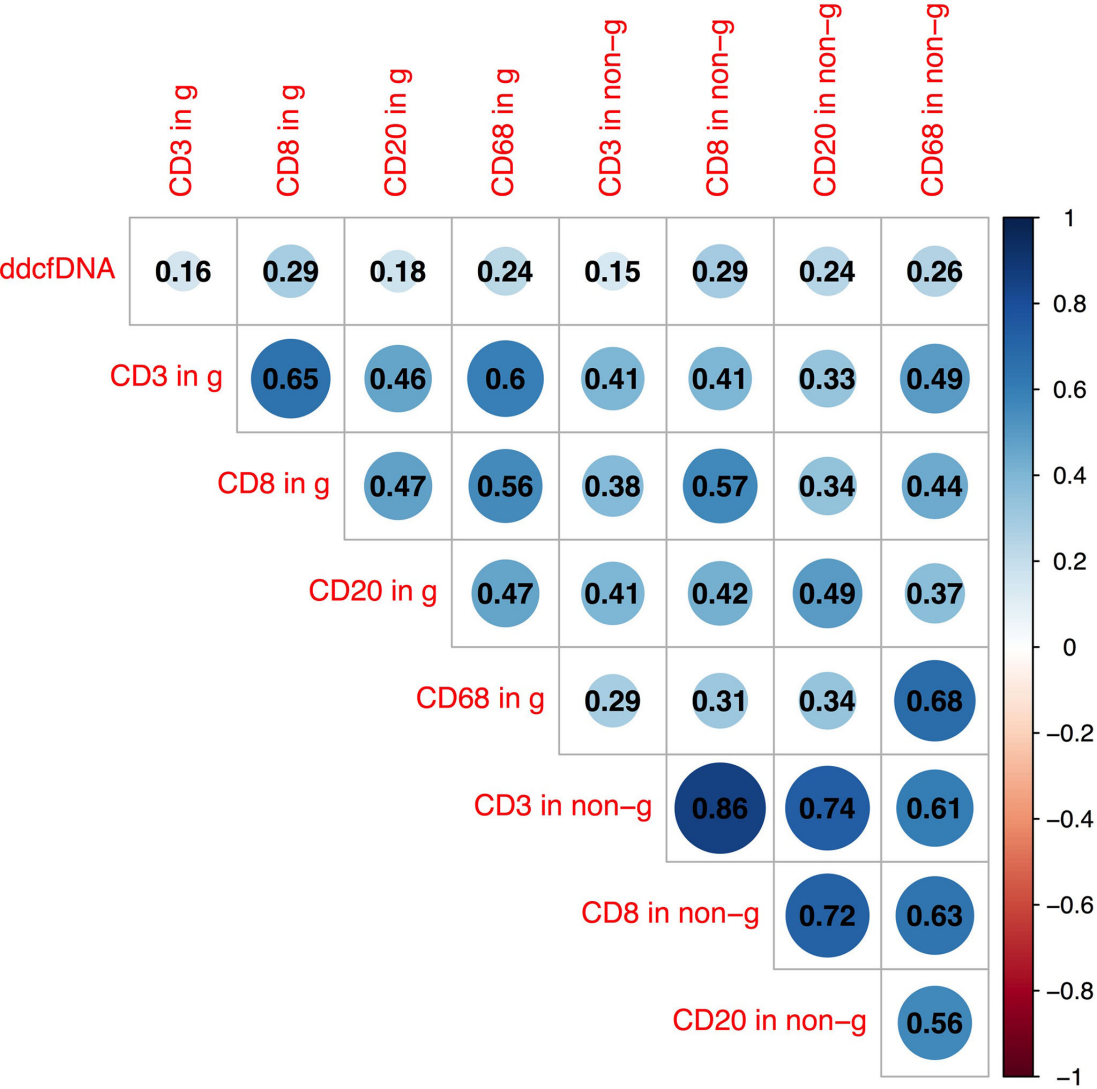
Pearson correlation analysis between plasma ddcfDNA fractions, T cell (CD3+), cytotoxic T cell (CD8+), B cell (CD20+), and macrophage (CD68+) infiltration in glomerular as well as non-glomerular area of corresponding biopsies. Numbers in triangle represent correlation coefficients (r). Blue bubbles represent positive correlations, while red bubbles represent negative correlations. g, represents glomerular area; non-g, non-glomerular area.

In previous analysis, we found that inflammatory cell infiltration in the allograft was correlated with higher plasma ddcfDNA levels, especially when endothelium was involved. In our study, recipients were divided into high (n = 35), medium (n = 36), and low ddcfDNA (n = 35) cohorts based on their plasma ddcfDNA fractions. The inflammatory infiltrates in biopsy tissues were phenotyped by immunohistochemical staining to further ascertain whether inflammatory infiltrate immune cell types in allografts correlated with plasma ddcfDNA levels in kidney transplant recipients. Immunohistochemical staining with CD3, CD8, CD20, and CD68 demonstrated the clustering of T cells, cytotoxic T cells, B cells, and macrophages in allograft biopsy areas. The density of CD3-, CD8-, CD20-, and CD68-positive inflammatory cells in the glomerular as well as non-glomerular areas was measured as described in the *Materials and Methods* section (**Supplementary Figure S4**). Average-linkage algorithm was applied to generate a dendrogram showing the hierarchical clustering of biopsies with different histopathologies, corresponding plasma ddcfDNA levels and infiltrated inflammatory cells (**Figure 3**). As shown in **Figure 3**, the plasma ddcfDNA levels in recipients tended to cluster according to the infiltrated inflammatory cell type. Pearson correlation test was applied to identify the relationship between plasma ddcfDNA fractions and the density of CD3-, CD8-, CD20-, and CD68-positive inflammatory cells in allografts (**Figure 4**). The densities of cytotoxic T cells (CD8 positive) and B cells (CD20 positive) in non-glomerular areas were positively correlated with plasma ddcfDNA levels in kidney transplant recipients (*P* = 0.01). Additionally, a significant positive correlation was found between macrophages in both glomerular and non-glomerular areas and plasma ddcfDNA levels, indicating that macrophage infiltration might contribute to severe allograft injuries (*P* ≤ 0.02).

### 3.4 Macrophage Extracellular Traps and Plasma ddcfDNA Fractions

**Figure 5:**
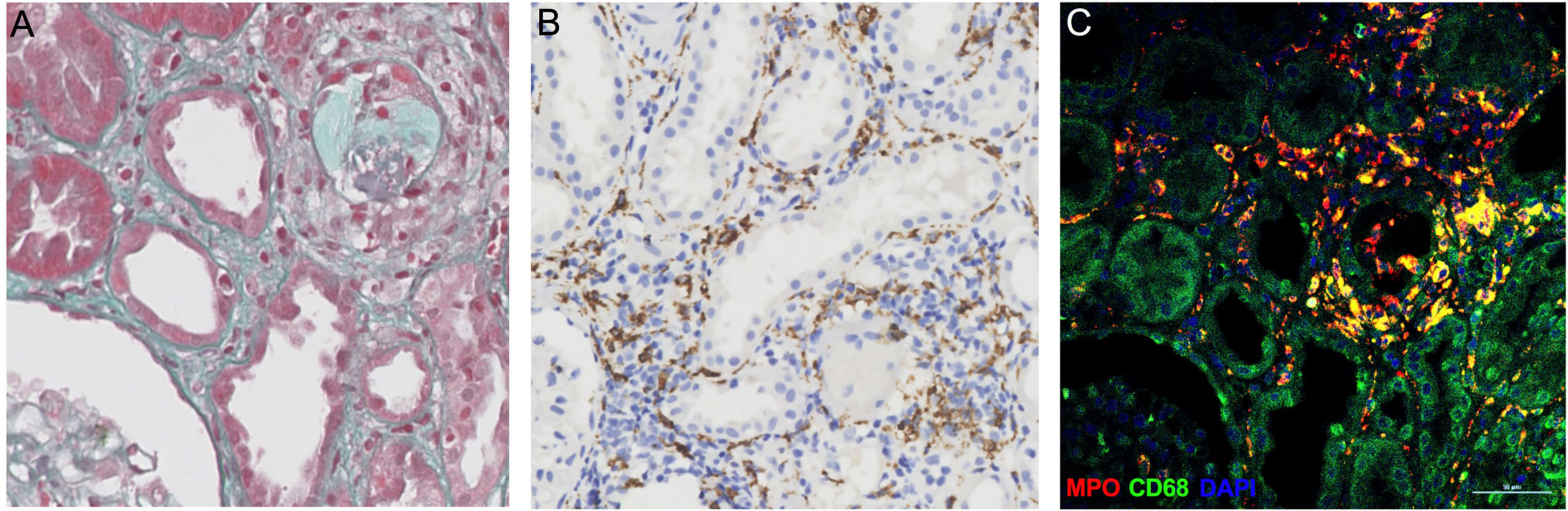
Myeloperoxidase (MPO)-containing macrophages in allograft biopsy of kidney transplant recipients. **(A)** Representative examples of Masson’s trichrome staining image of allograft biopsy. **(B)** Immunochemistry staining with anti-CD68 antibodies for macrophages in allograft biopsy. **(C)** Immunofluorescence images to show colocalization of MPO+ (in red) with macrophages (CD68+, in green). Scale bar: 50 μm.

**Figure 6:**
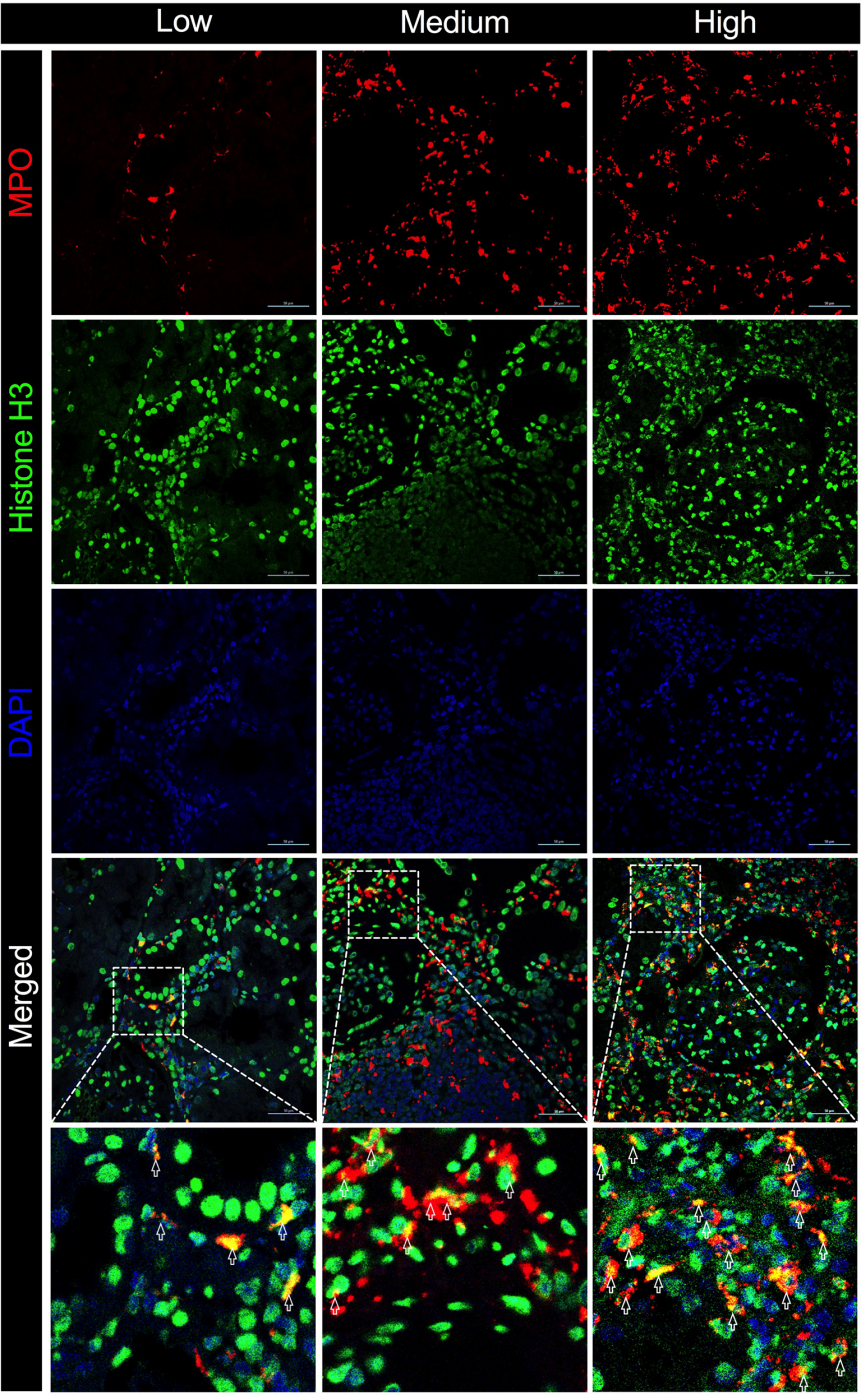
Macrophage extracellular traps (METs) in allograft biopsies of kidney transplant recipients associated with elevation in plasma ddcfDNA levels. Immunofluorescence staining with anti-MPO (in red) and anti-Histone H3 (in green) antibodies for METs in allograft biopsy. Representative images of METs in low (ddcfDNA 0.38%, TCMR IB), medium (ddcfDNA 0.87%, TCMR IIA), and high (ddcfDNA 9.99%, ABMR) plasma ddcfDNA-level cohorts. Boxed areas in the merged images present the regions of interest showing double positive cells in light yellow. Arrows are typical examples for METs. Scale bar: 50 μm.

Macrophages form a vital component of innate immunity, which have multiple functions ranging from tissue repair and homeostasis maintenance to immune surveillance. The abundance of macrophages in allograft has been documented to be associated with allograft rejection (25). Recent studies have demonstrated that macrophages were capable of generating ETs that contribute to apoptosis in kidney diseases (19). In the present study, the immunofluorescence analysis of allograft biopsies with prominent macrophage infiltrations exhibited notable colocalization of CD68 and MPO (**Figure 5**), confirming that the majority of plausible ETs in allograft were mostly produced by MPO-containing macrophages (METs). Accordingly, METs were characterized as double-positive for MPO and Histone H3 cells in allograft biopsies to further evaluate the correlation between MET activity and plasma ddcfDNA (**Figure 6**). Immunofluorescence staining confirmed the presence of METs (MPO+H3+ cells) in glomerular and peritubular capillary areas of biopsies among low, medium, and high plasma ddcfDNA subgroups (**Figure 6**). Additionally, as shown in **Figure 6**, the presence and extent of METs were positively correlated with increased plasma ddcfDNA levels. In a nutshell, our results demonstrated that the infiltration of macrophages in allograft probably contributed to releasing ddcfDNA by METs.

## 4 Discussion

Routine measurement of serum creatinine levels is the most prevalent method to monitor allograft function after kidney transplantation. However, the clinical utility of serum creatinine is significantly limited by its low accuracy. A study by Yang et al. (26) reported that nearly 40% of indication biopsies had stable outcomes or presented with no injury, while 40% of allograft injuries were observed in protocol biopsies. Importantly, the elevation of plasma ddcfDNA levels, derived from damaged allograft cells, has been documented to be associated with allograft rejection (especially ABMR), infection, and *de novo*/recurrence of glomerulonephritis (27), albeit the observed heterogeneity in ddcfDNA levels. Recent investigations have reported that increased plasma ddcfDNA levels are associated with intrinsic factors like recipient body mass index, age, and tacrolimus concentration (5, 28). Yet, the mechanisms underlying this heterogeneity have not been fully unraveled, which has limited the clinical application of ddcfDNA.

Herein, we observed that plasma ddcfDNA levels in the TCMR subgroup were rather fluctuated, which might be associated with the histopathological diversity of TCMR in Banff classification. Consistent with findings of a study by Cheng et al. (29), we previously analyzed the ddcfDNA levels in the subgroups of TCMR and observed an elevation of circulating ddcfDNA levels in TCMRs with intimal arteritis (unpublished). Active ABMR and type II/III TCMR share similar microvascular injury phenotypes and presented with comparable plasma ddcfDNA levels, suggesting that histological lesions potentially participate in the mechanisms underlying ddcfDNA elevation. Interestingly, Gielis et al. (8) found that the presence of ptc rather than i, t, g, v, or c4d was associated with increased ddcfDNA fractions. Nevertheless, due to the limited number of allograft rejections, obvious histopathological subelements that were more strongly associated with ddcfDNA elevation remain uncertain. Accordingly, in this study, we sought to extend the clinical value of ddcfDNA by investigating the association between the increment in ddcfDNA and Banff histopathological subelements. Our results demonstrated that g, v, and mvi were strongly correlated with elevated plasma ddcfDNA fractions and concentrations. Microvascular inflammation is the typical histological manifestation of ABMR, which could explain the elevated plasma ddcfDNA levels with increased g and mvi scores.

However, in contrast with the study by Gielis et al. (8) , we found that ptc and t were not significantly correlated, which may be accounted for by the limited specificity of ptc for ABMR. The incidence of Banff lesion score ptc and t has been reported to be higher than 10% in TCMR and borderline changes (30). Importantly, the Banff lesion score ptc is based on the number of inflammatory cellular infiltrate in the most severe peritubular capillary, reflecting localized inflammatory severity rather than inflammation in the whole biopsy specimen. In contrast, Banff lesion score g is determined by the proportion of glomeruli involvement in allograft biopsies, which could be a better histopathological subelement than ptc regarding acuity of allograft injury caused by immune system activation. In the present study, we also observed that an increase in i score was accompanied by elevated plasma ddcfDNA fractions. Interestingly, changes in cfDNA concentration have been reported to be associated with the infarct volume following focal ischemic injury in rat models (31) and also correlated with the area as well as severity of burn injury in burn patients (32), which illustrated that cfDNA has potential clinical value in reflecting the severity of injury. Therefore, it can be inferred that increments in plasma ddcfDNA levels may be proportional to the extent and severity of allograft injury, especially with vascular injuries such as g and v. These findings suggested that changes in circulatory ddcfDNA levels were accounted for not only by the allograft injury type but also by the inflammatory microenvironment, which induced increased permeability and extravasation of cfDNA through pores in the vasculature into the circulation. However, the underlying mechanism remains unclear, warranting further studies.

Cellular infiltrations in allografts consist of a variety of immune cells that participate in allograft injury. A study based on single-cell RNA analysis of biopsy specimens from patients undergoing allograft rejection revealed the relevance between rejection and monocyte/macrophage type (33). Sablik et al. (34) reported that a predominance of infiltrating CD8+ T cells in chronic active ABMR and increased interstitial FoxP3+ T cells were risk factors of allograft failure. Immunohistochemistry staining was adopted to visualize immune cell infiltrates in the allograft. Additionally, digital image recognition was applied to count positive staining cells and determine their impact on plasma ddcfDNA levels in allograft recipients. Hierarchical clustering and Pearson correlation analysis revealed a significant positive correlation between cytotoxic T cells (CD8 positive), B cells (CD20 positive) in the non-glomerular area and plasma ddcfDNA levels in kidney transplant recipients. These results suggested that the ddcfDNA levels also reflected the severity of some types of allograft injuries. The difference might be attributed to the heterogeneous injury mechanisms and cell types involved. Especially, we found that macrophages in glomerular and non-glomerular areas were significantly correlated with plasma ddcfDNA levels, indicating that macrophage infiltration might contribute to severe allograft injuries.

The mechanism of cfDNA released in the blood circulation has not been fully documented. Apart from allograft damage, necrosis, and apoptosis, recent investigations have revealed NETosis as another important origin of cfDNA (35). Notwithstanding that most studies have found that cfDNA were neutrophil-derived, other immune cells, including macrophages, could also generate ETs (36–38). The formation of METs is highly analogous to ETosis generated by neutrophils (39). Similar to NETs, the activity of METs was also reportedly induced by inflammation in sterile environments in the absence of active infection (40–42). Consequently, we hypothesized that METs were involved in the pathogenesis of allograft injury. Abundant macrophage infiltration was visualized in allograft biopsy tissues by immunochemistry staining of CD68 (**Figure 5**). The immunofluorescence assay revealed that CD68 and MPO [an important lysosomal enzyme previously reported to be released by ETs (11)] were colocated in biopsy tissues. Moreover, O’Sullivan et al. (19) showed that the most active glomerular lesions correlated with MPO+ macrophage infiltration in the intraglomerular area. Given that MPO is prominently expressed in macrophages, MPO and Histone H3 double-positive cells were characterized as macrophages undergoing ETosis (METosis) in biopsy samples. The immunofluorescence assay demonstrated the significant presence of METs in glomerular and peritubular capillary areas of biopsies with high plasma ddcfDNA levels, while minor MET infiltrations were observed in biopsies with low plasma ddcfDNA levels.

METosis has been documented as a rapid process occurring less than 30 min (43, 44), which possibly contributes to the high turnover and rapid clearance rate of cfDNA (45, 46). Despite participating in the innate immunological process, persistent ETs could also provoke local inflammation, resulting in the activation of adaptive immunity (39), which mediated pathogenesis in a variety of autoimmune diseases, including psoriasis (47), type I diabetes (48), and rheumatoid arthritis (49). Furthermore, Lande et al. (50) reported that ET-derived factors triggered B-cell activation in systemic lupus erythematosus, inducing secretion of autoantibodies by plasma cells. Although NETs have predominantly been documented in the literature, recent studies have reported that METs were also an important form of ETs that might have similar functions to NETs. Accordingly, we hypothesize that METs could digest injured allograft cells and release ddcfDNA through vessels in allografts. Moreover, the formation of METs could probably induce activation of the humoral immune system in recipients for secretion of donor-specific antibodies leading to ABMR, which could be explained to a certain extent by the significant elevation of plasma ddcfDNA levels in ABMR in our study (2).

Some limitations were present in our study. First, the sample size was relatively small; accordingly, we could not unravel the relationship between plasma ddcfDNA levels and other Banff lesions such as mm, ah, and ahh due to their low incidence in allograft biopsies. Moreover, the underlying mechanism of METs in kidney transplantation was not fully determined, emphasizing the need for more studies to uncover the impact of METs on allograft injury and donor-specific antibodies.

Herein, we observed that changes in plasma ddcfDNA levels could reflect the severity of allograft injury and the inflammatory burden in allografts. Correlation studies and immunofluorescence assay revealed that METs in allografts, especially microvascular areas, were associated with increased plasma ddcfDNA levels. Our findings suggest that METs might contribute to the activation of inflammation during allograft injury and possibly contributed to the release of ddcfDNA from damaged allograft cells. The documented correlation between Banff lesion scores and ddcfDNA helps broaden our understanding of how liquid biopsy assay can provide information on allograft status.

## Data Availability Statement

The datasets presented in this study can be found in online repositories. The names of the repository/repositories and accession number(s) can be found below: https://db.cngb.org/cnsa/, CNP0002302.

## Ethics Statement

The studies involving human participants were reviewed and approved by the Research Ethics Committee of the First Affiliated Hospital of School of Medicine, Zhejiang University. The patients/participants provided their written informed consent to participate in this study.

## Author Contributions

LG and RW contributed to the study design and data interpretation. LG, JS, and RW contributed to data analysis, data interpretation, and writing. WL, PY, MW, and QZ contributed to data collection. HW, JW, and JC contributed to article writing advice. All authors contributed to the article and approved the submitted version.

## Funding

This study was financially supported by the Science and Technology Department of Zhejiang Province (Grant No. 2019C03029), Bethune Charitable Foundation (grant number, G-X-2019-0101-12), and the National Natural Science Foundation of China (Grant Nos. 81870510, 81770719, and 82070766).

## Conflict of Interest

The authors declare that the research was conducted in the absence of any commercial or financial relationships that could be construed as a potential conflict of interest.

## Publisher’s Note

All claims expressed in this article are solely those of the authors and do not necessarily represent those of their affiliated organizations, or those of the publisher, the editors and the reviewers. Any product that may be evaluated in this article, or claim that may be made by its manufacturer, is not guaranteed or endorsed by the publisher.
