# Supplementary material for: Plasma Donor-Derived Cell-Free DNA Levels Are Associated With the Inflammatory Burden and Macrophage Extracellular Trap Activity in Renal Allografts
Source: Front Immunol. 2022 Mar 21;13:796326. doi: 10.3389/fimmu.2022.796326 (PMC8977515; doi:10.3389/fimmu.2022.796326)
Supplement: Supplementary file 1 [file DataSheet_1.pdf]

# Supplementary Material

## 1 Supplementary Figures and Tables

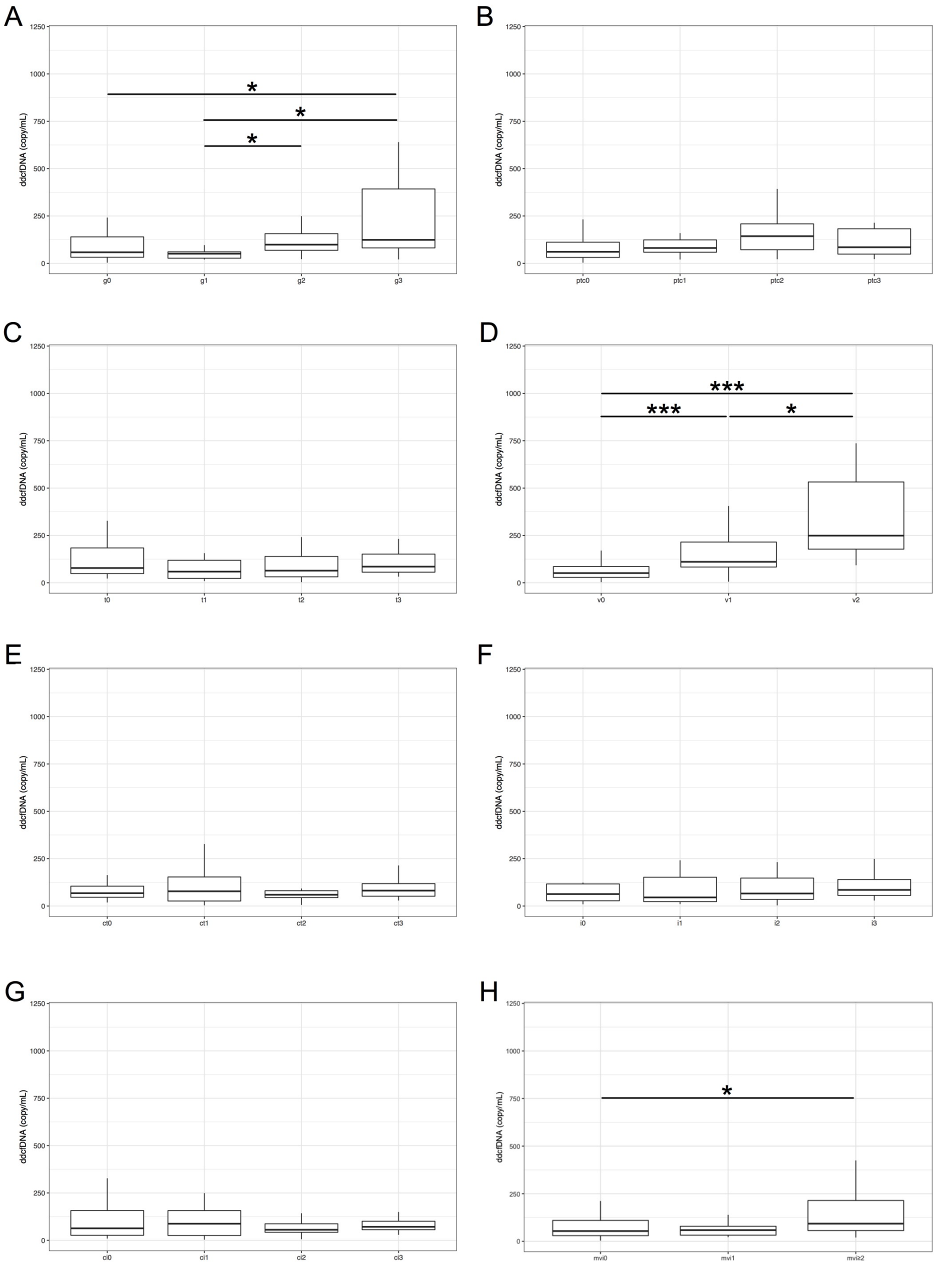


**Supplementary Figure 1.** Plasma ddcfDNA concentrations in kidney transplant recipients with different Banff lesion scores. (A) glomerulitis (g); (B) peritubular capillaritis (ptc); (C) tubulitis (t); (D) intimal arteritis (v); (E) tubular atrophy (ct); (F) interstitial inflammation (i); (G) interstitial fibrosis (ci); and (I) microvascular inflammation (mvi, g+ptc). Boxplot with bold line represents median levels. * $P < 0.05$, ** $P < 0.01$, and *** $P < 0.001$.

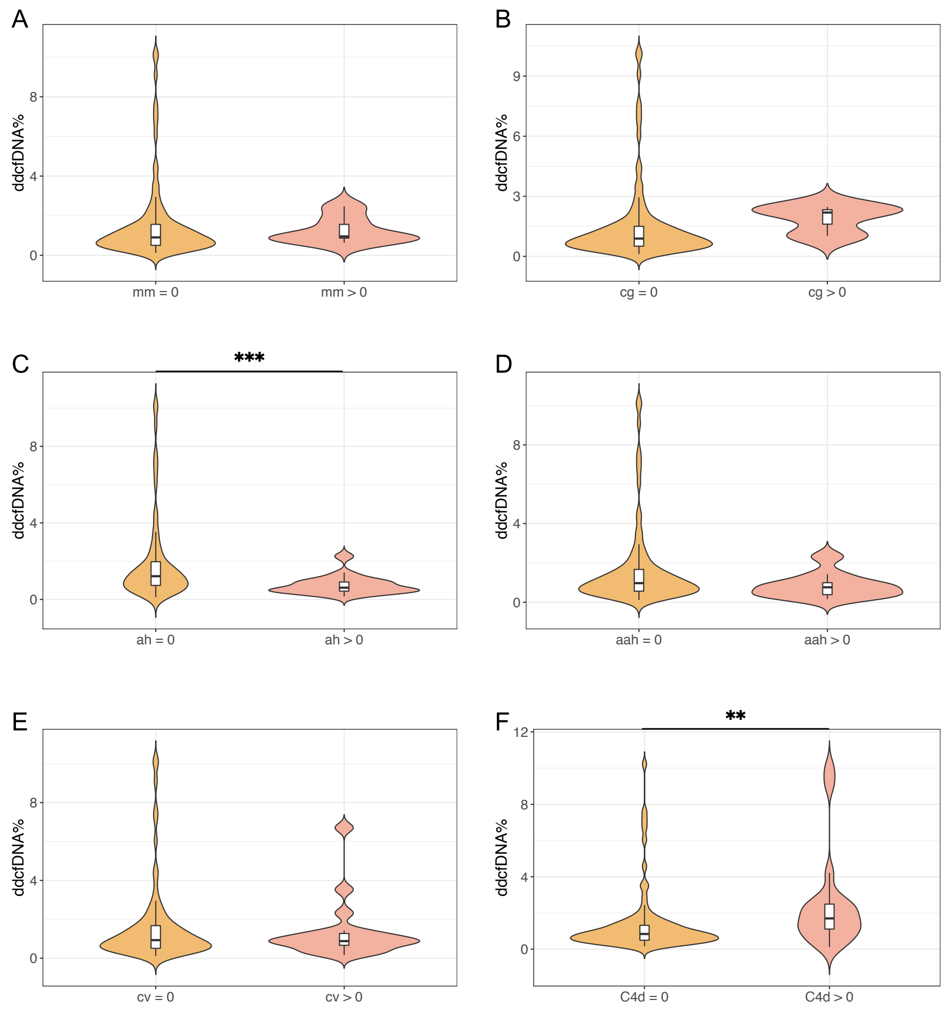


**Supplementary Figure 2.** Plasma ddcfDNA fractions in kidney transplant recipients with different Banff lesion scores. (A) mesangial matrix (mm); (B) GBM double contours (cg); (C) arteriolar hyalinosis (ah); (D) arteriolar thickening (aah); (E) arterial intimal fibrosis (cv); (F) C4d; Violin plot with bold line represents median levels and box indicating the interquartile range. ** $P < 0.01$, and *** $P < 0.001$.

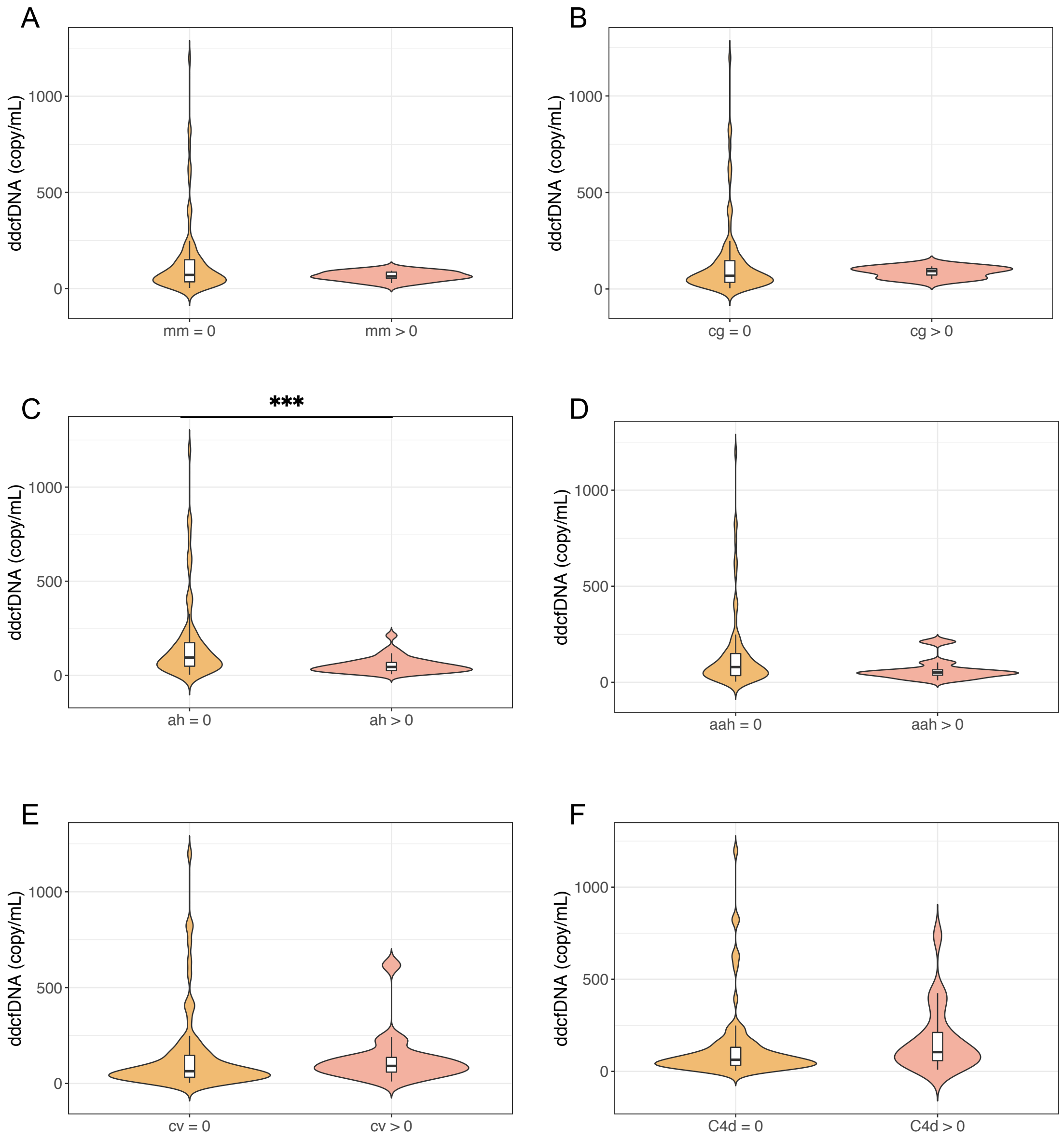


**Supplementary Figure 3.** Plasma ddcfDNA concentrations in kidney transplant recipients with different Banff lesion scores. (A) mesangial matrix (mm); (B) GBM double contours (cg); (C) arteriolar hyalinosis (ah); (D) arteriolar thickening (aah); (E) arterial intimal fibrosis (cv); (F) C4d; Violin plot with bold line represents median levels and box indicating the interquartile range. ** $P < 0.01$, and *** $P < 0.001$.

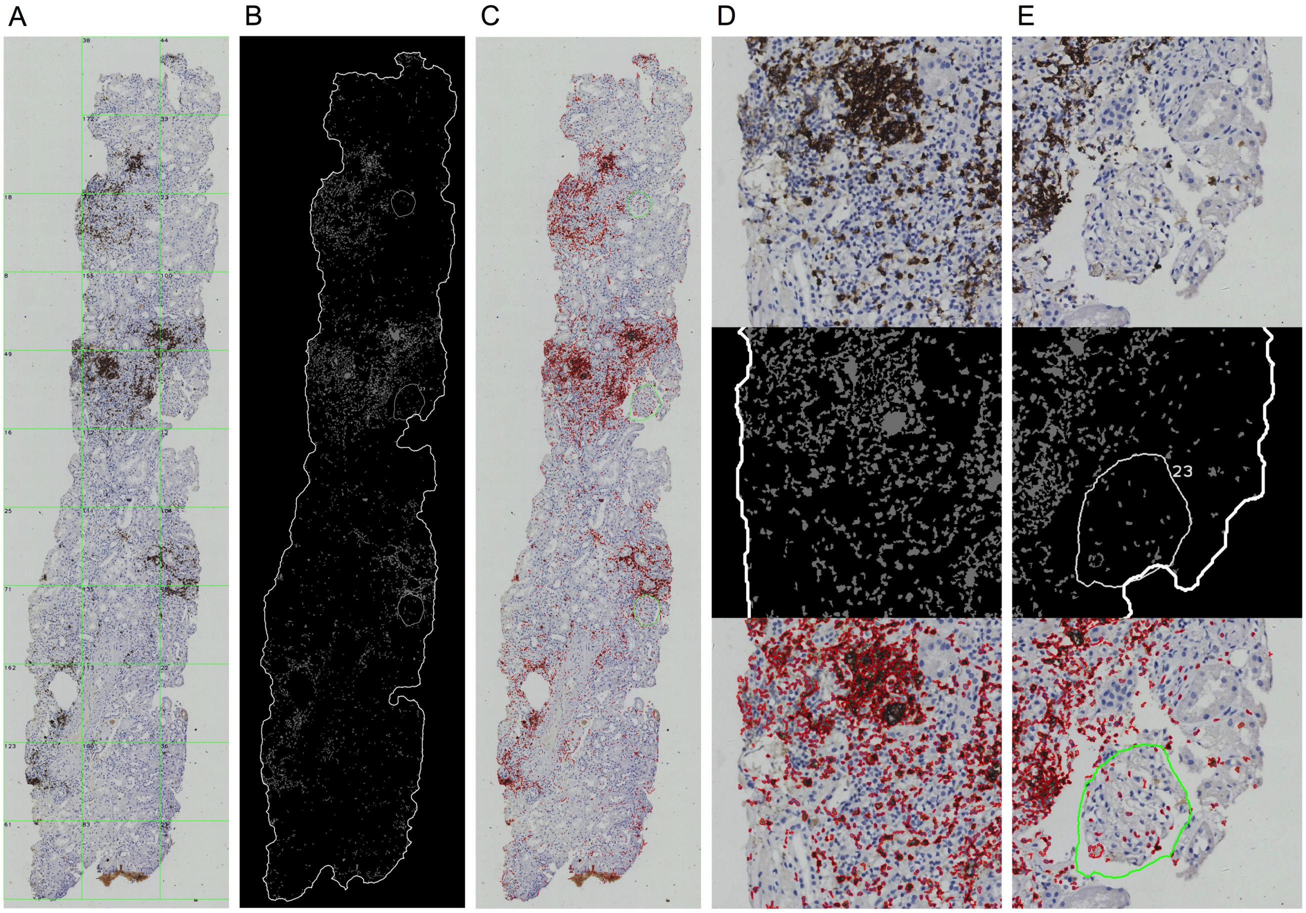


**Supplementary Figure 4.** Representative image of digital image recognition. (A) grid image; (B) binary image; (C) merged image; general overview of cell (D) and glomeruli (E) segmentations (20X).
